# Re-introducing the Cambridge Group Family Reconstitutions

**DOI:** 10.51964/hlcs9311

**Published:** 2020-09-22

**Authors:** George Alter, Gill Newton, Jim Oeppen

**Affiliations:** University of Michigan, USA; University of Cambridge, UK; University of Southern Denmark, Denmark

**Keywords:** Historical demography, Fertility, Family reconstitution, Passive registration

## Abstract

*English Population History from Family Reconstitution 1580–1837* was important both for its scope and its methodology. The volume was based on data from family reconstitutions of 26 parishes carefully selected to represent 250 years of English demographic history. These data remain relevant for new research questions, such as studying the intergenerational inheritance of fertility and mortality. To expand their availability the family reconstitutions have been translated into new formats: a relational database, the Intermediate Data Structure (IDS) and an episode file for fertility analysis. This paper describes that process and examines the impact of methodological decisions on analysis of the data. Wrigley, Davies, Oeppen, and Schofield were sensitive to changes in the quality of the parish registers and cautiously applied the principles of family reconstitution developed by Louis Henry. We examine how these choices affect the measurement of fertility and biases that are introduced when important principles are ignored.

## 1 INTRODUCTION: THE CAMBRIDGE GROUP RECONSTITUTIONS

In their 1997 book *English Population History from Family Reconstitution 1580–1837*, Wrigley, Davies, Oeppen and Schofield of the Cambridge Group for the History of Population and Social Structure (CAMPOP) describe a dataset compiled from record linkage of Anglican church baptism, marriage and burial records for 26 parishes situated around England. Since the family reconstitution volume was published, research in historical demography has expanded and new methods, like event history analysis, are now standard (Alter, 2019). These data are an important resource, and we believe that they will remain a valuable resource for future generations of researchers. This document is intended as a guide for researchers who use the database. We describe the process of migrating the data from the original paper forms that were digitized into structured text files, later translated into a relational database; and from the relational model into the Intermediate Data Structure (IDS).

*English Population History from Family Reconstitution 1580–1837* was one of the last large scale studies based on the classic approach to family reconstitution data, and it shares a number of features with similar work in France (Henry, 1956, 1972a, 1972b, 1978; Henry & Blayo, 1975; Houdaille, 1976; Henry & Houdaille, 1973), Germany (Knodel, 1988), and Quebec (Charbonneau, 1975; Henripin, 1954). It was primarily concerned with establishing levels and trends and describing key demographic patterns, such as the impact of age at marriage on fertility. Analysis of differentials by occupation, literacy, or religion is limited. This volume was also viewed as refining and continuing work on national level demographic parameters determining England’s past population growth first addressed in *The Population History of England, 1541–1871: A Reconstruction* (Wrigley & Schofield, 1981), and family reconstitution results were compared to estimates from Back Projection and new estimates from Generalized Inverse Projection. The analysis relies on descriptive statistics (means, rates) rather than statistical models, such as regression and event history analysis. This is not meant to imply that the volume was in any way naïve or basic. On the contrary, its methodology was innovative and displayed a sophisticated understanding of demographic models that is often lacking in later research in historical demography (Alter, 2019).

There are many opportunities for important and innovative research with these data. More than 100,000 conjugal families were reconstituted over a period from 1540 to 1850. Statistical tools, such as event history analysis, are much more available today, and they can be deployed to look at topics like demographic responses to short-term economic stress (Bengtsson, Campbell, & Lee, 2004; Lundh & Kurosu, 2014; Tsuya, Wang, Alter, & Lee, 2010). There are also important opportunities for studying intergenerational transmission of mortality, fertility, and marriage. The database contains 66,027 lineages with three generations in the female line (grandmother-mother-child), 20,348 female lineages with four generations, and 6,645 female lineages with five generations.

*English Population History from Family Reconstitution* devoted enormous attention to data quality and representativeness. The volume includes ten appendices, most of which are devoted to methodological issues, and entries for ‘reliability of registers’ cover about a column and a half in the Subject Index. One of our goals is to highlight and explain these concerns to future users of these data. We emphasize decisions about data selection that affect analyses. In particular, we show that ignoring key rules about family reconstitution result in biased samples and misleading results.

This paper has two parts. Section 2 describes the CAMPOP family reconstitutions and gives a history of their representation in different digital formats. We describe how the reconstitutions travelled from their initial digital form on paper tape to a relational database and the entity-attribute-value system used in IDS. This section will be especially useful to researchers who plan to work with the data in a relational database, or are using the original UK Data Archive deposited versions of these data (previously available directly from CAMPOP) which are delimited text tables output from an Access relational database. It also provides background on the original mode of representation and precision/fuzziness of key variables, which affect all translations of these complex, highly-derived data, and the provenance and limitations of available metadata.

Section 3 uses the CAMPOP family reconstitutions to examine a fundamental issue in all family reconstitution research: Which families should be included in the analysis of marital fertility? Many different considerations, such as the last observed event and the precision of reporting of dates, affect whether a family can be included in a particular analysis. We calculate and compare age-specific fertility rates under a number of different selection rules. Some rules, such as the Henry criteria for identifying complete fertility histories, have important implications for results. The Fleury and Henry manuals for family reconstitution (Fleury & Henry, 1956, 1965, 1985) were based on important principles of event history analysis that were well understood by researchers at the Cambridge Group. We recommend this part to anyone who plans to use the CAMPOP database or any other family reconstitution data.

## 2 HISTORY OF THE CAMPOP FAMILY RECONSTITUTION DATABASE

### 2.1 SHARING FAMILY RECONSTITUTION DATA

Even though large collections of family reconstitution data for England, France, Germany, and other places have been available, they have been under-utilized by subsequent researchers. Family reconstitution was invented as a set of operations performed with paper and pencil. Henry and others used computers to analyze the data, but no standard format for analyzing or storing family reconstitutions emerged. This hindered sharing of data and analytical procedures. In the absence of a standard data model computer programs for analyzing family reconstitutions were re-invented many times.

Family reconstitution data are inherently complex, and they are not easily converted into a rectangular data structure, such as a spreadsheet or statistical analysis file. Families vary in size from 0 to 20 children, and the amount of information on each person (dates of birth, marriages, death; residences; occupations) differs. Each person belongs to one family by birth but may form several families through marriage. Family reconstitution data also record kinship relationships that may extend across ten generations, primarily focused in each generation on nuclear family members. The sources used in family reconstitution may include additional information that is specific to a place and time. For example, the Napoleonic Code required four witnesses to every marriage, who are often described by occupation and relationship to the bride and groom. In Latin America the names recorded in parish registers provide clues to the process of cultural change (Thurtell & Klancher Merchant, 2018)

The Intermediate Data Structure (IDS) was created to facilitate sharing of longitudinal historical demographic data and to increase transparency in methods used to analyze these data (Alter & Mandemakers, 2014). IDS is extremely flexible, and it can be extended to accommodate almost any kind of demographic and social information. IDS promotes both sharing data and re-using computer code by providing a standard that can be applied to many different databases. There is also a cost to flexibility, because IDS requires a layer of programming to produce ‘episode’ files suitable for analysis by standard statistical analysis software. Our hope is that researchers will share and re-use these ‘extraction programs’, like the software used in this paper. For example, Quaranta and Sommerseth (2018) used the same computer code to analyze IDS data from Belgium, the Netherlands, Norway, and Sweden.

IDS is not intended to be the format in which the data are analyzed. Data for event history analysis in some statistical packages, like Stata, must be organized into ‘episodes’. Each episode describes a segment of time in which the independent variables used for analysis remained constant. An episode may start and end with observed events (e.g. a birth, death, or marriage), or be ‘right censored’ if the closing event happens after observation ends, ‘left-truncated’ if the event that starts an episode happened before observation begins, or both. As we discuss in Section 3, the rules for selecting which families and episodes can be included for analysis are complex and depend upon the objectives of the research.

### 2.2 STRUCTURE OF THE ORIGINAL DATASET RECORDS

The Cambridge Group family reconstitutions were created by the classic method popularized by Fleury and Henry (1956). Baptisms, marriages, and burials were transcribed from parish registers to slips of paper, which were then sorted into families. Volunteer historians recorded information on slips of paper and then combined families on Family Reconstitution Forms (FRFs), which are the source of the digital data that we have today (Wrigley, Davies, Oeppen, & Schofield, 1997, pp. 563–568). We describe here the steps in moving from paper to digital files and into a relational database. Initially there were separate structured text files for each of the 26 parishes, which were converted to relational database structure and ultimately harmonized into one Microsoft Access database. This was undertaken in 2003 and 2013 by Gill Newton at CAMPOP.

**Figure 1:**
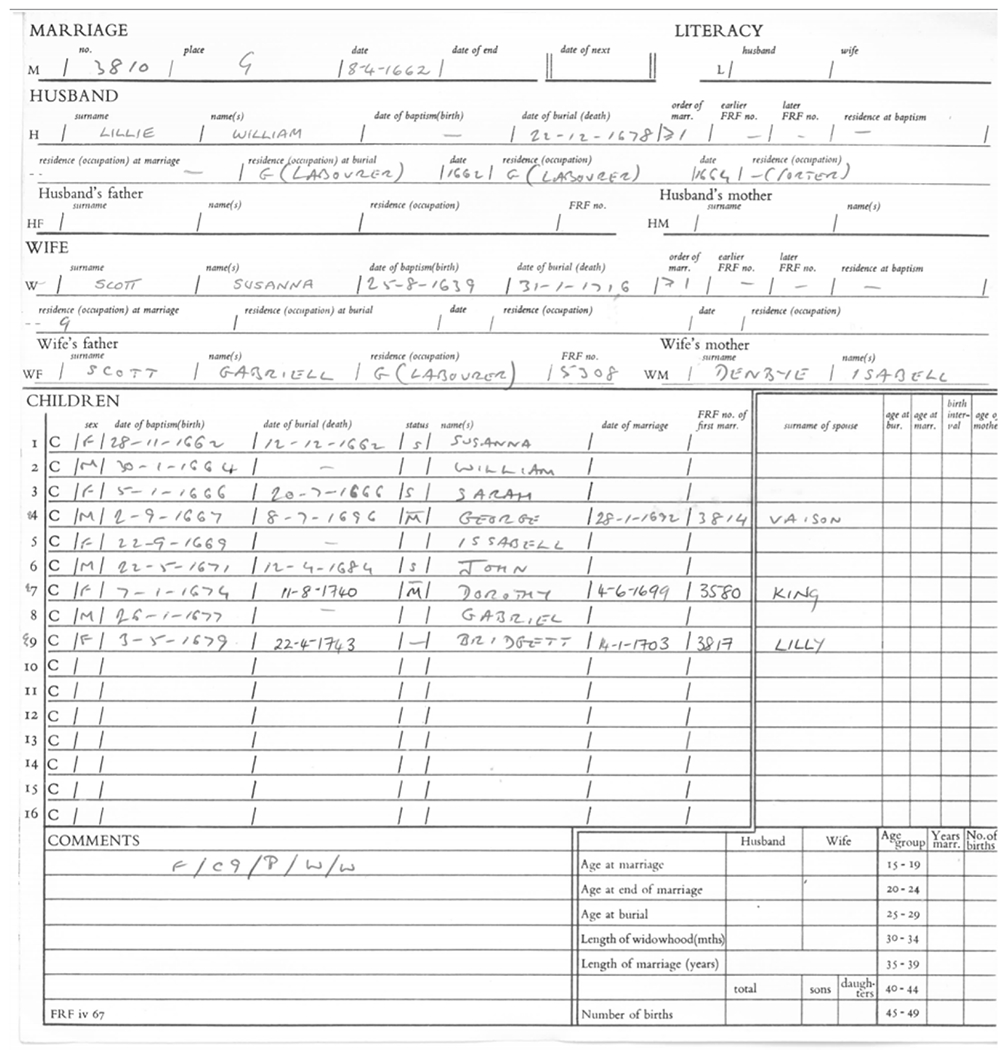
Example paper Family Reconstitution Form (FRF) record

An example handwritten paper Family Reconstitution Form (FRF) is illustrated in Figure 1. These *pro forma* paper sheets are divided into separate regions, each containing information filled in by hand on individual family members who might be named in parish records. Central to each FRF is the marriage record, either actual or inferred, which is the start point of a reconstituted nuclear family.

FRFs were compiled by hand from precursor baptism, marriage and burial slips containing pertinent information transcribed direct from events recorded in church registers (or taken from printed transcripts of such registers published by genealogical societies), so that each FRF represents a distillation of potentially several sources of information on the same individuals. For further information on the process of manual record linkage and compiling the information see Wrigley (1966); see also Newton (2011) for a description of the evolution of family reconstitution record linkage methods at CAMPOP.

Textual and numeric data from the FRFs were originally punched onto paper tape from 1967 onwards in either KDF9 8 channel code or Ferranti Mercury 5 channel code, for processing by the computing laboratory at the University of Newcastle-upon-Tyne.^1^ The data were structured in GENDATA format, a flexible data input and record management system developed at Cambridge and compliant with the Newcastle File Handling System.

An FRF record was represented by a number of lines of textual information appearing in a particular order. Each line was composed of a number of data-containing parts separated by a forward slash (/). Core variables in each line class were always represented, and absent values were indicated by dash or hyphen (−). Both the number of variables per line and the number of lines per record could vary.

The class of information recorded on each line was indicated by initial character variables. Thus, M/ at the beginning of the line denoted marriage information; H/ denoted information on the husband; W/ information on the wife; and C/ information on a child. Each information line class was drawn from one region of the handwritten paper FRF *pro forma*. Some family reconstitutions had more line classes than others. Additional lines might represent literacy (inferred from husband and wife’s ability to sign the marriage register). Some but not all family reconstitutions had information on the parents of the married couple (taken from suitable marriage registers or their own baptisms), and thus additional HM/ line classes denoting husbands’ mothers, HF lines denoting husbands’ fathers, and similarly WM/ and WF/ lines for wives’ parents.

Most line classes appeared once per record, representing a single marriage, husband, wife, husband’s mother, husband’s father, wife’s father or wife’s mother. Some line classes repeated as multiple instances in the same record, as with child lines where a marriage produced more than one child.

Each record was given a unique identifying number, represented as the first variable after the initial class code of the first line of the record, which was always the Marriage line. Child lines were ordered but lines within a record were not otherwise numbered. In Flag lines, which give additional information on demographically significant characteristics of particular individual(s) in a family, specific instances were referenced using the class code in combination with the order in which the line appeared, so for example C2 references the second Child line.

Some variables within lines were repeated, typically in pairs, for example to represent multiple additional dates and places of residence as part of a Husband or Wife line (such dates were typically sourced from information in the baptism or burial records of their children). Flag lines were composed entirely of a number of repeating pairs of variables. The first half of a flag variable pair indicated which line class instance within the record the extra information applied to, and the second half of the pair represented the coded value of the additional information, which could be compounded to represent multiple pieces of information. So for example the flag line F/H/W/C1/Z2/C2/Z2 indicates that a husband was widowed (flag value: W) and that the first and second child recorded in the same FRF record were stillborn (flag value: Z) and also twins (flag value: 2).

Some date variables within line classes represented pairs of dates within a single variable (as with baptism and birth date when both were known, or similarly burial and death date). In these cases the second date of the pair was enclosed in brackets, for example /15-7-1780 (21-6-1780)/. Uncertainty and other complications in date variables were indicated by numeric codes appended after the date and known as weights or weightings, although these should not be regarded as weights in the statistical sense. These weightings were indicated by leading stars or asterisks (*). So for example, /0-7-1780*101/ indicated a date known only to the month and represented as the earliest possible date, which is the meaning of weighting code 101.

**Figure 2:**
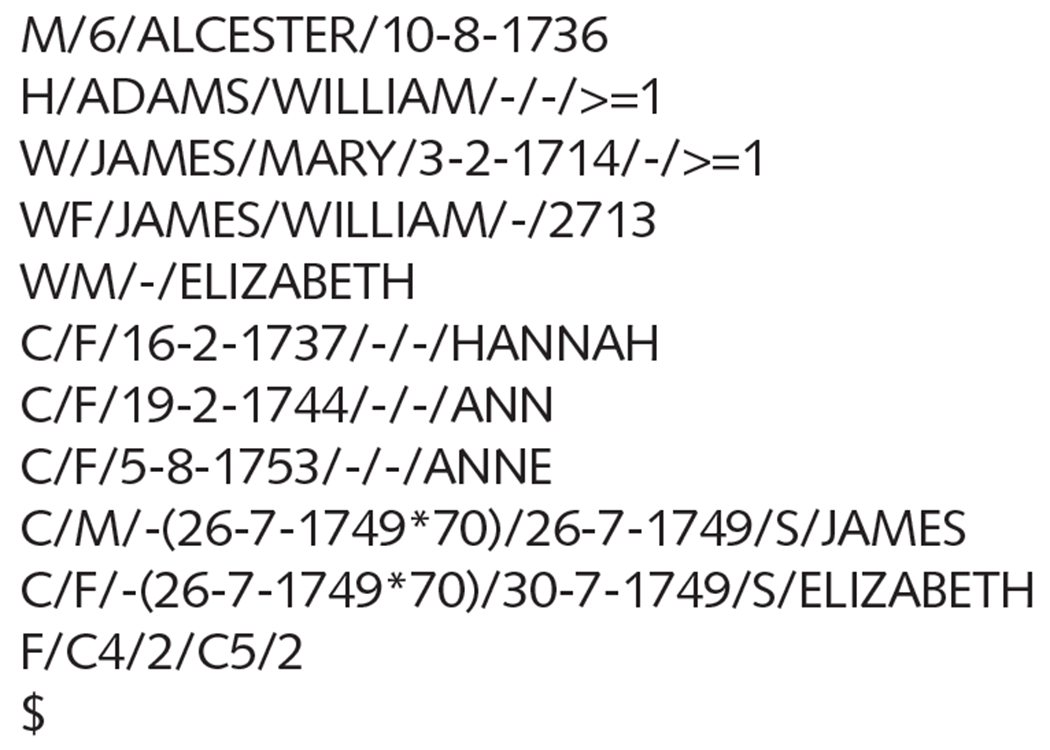
Example FRF record as originally digitised

**Figure 3:**
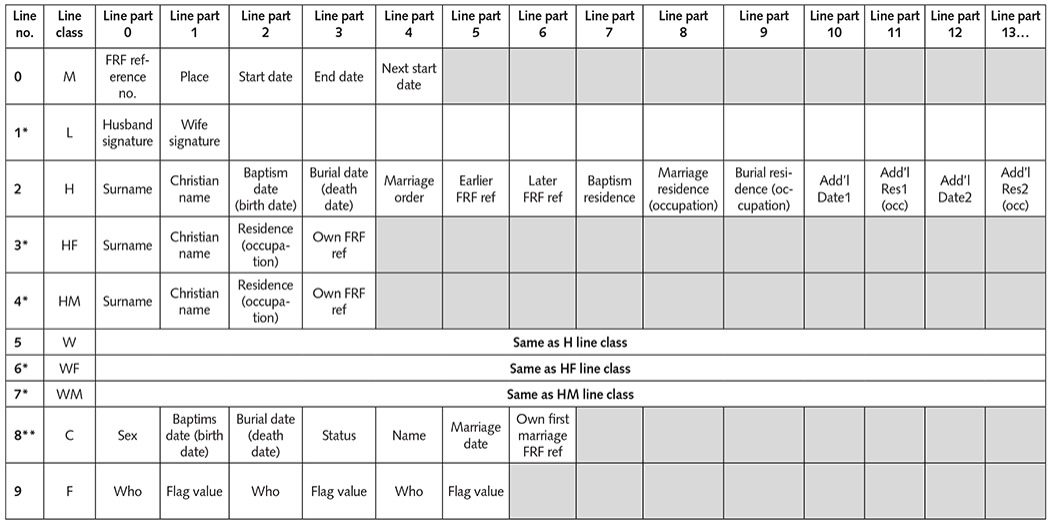
Schematic matrix representation of variable positions in original FRF digital record format NB: Line numbers are notional only since some line classes indicated by * are optional and others indicated by ** may occur many times in the same record. Key to line classes: M = Marriage L = Literacy H = Husband HF = Husband’s father HM = Husband’s mother W = Wife WF = Wife’s father WM = Wife’s mother C = Child F = Flag

After later digitisation of the punched paper tapes, the same structure described above was retained, with each multi-line record now terminated by the dollar sign symbol ($). An example FRF represented in this record format is shown in Figure 2. This formed the input for subsequent restructuring into relational database tables. Figure 3 gives a schematic matrix of the information each line and variable represents for FRF records in this original digital format.

### 2.3 CONVERTING THE DATA INTO RELATIONAL DATABASE TABLES

As each of the 26 family reconstitution databases differed slightly in format, each was processed separately. Initial attempts at a step-by-step transformation procedure proved too slow and cumbersome, so a program was written in Python to read in text files of FRF records, process them into the desired new data structure, and output delimited text files, one per proto-database table.

The guiding principle of the conversion was to lose no information while retaining as similar a data structure as possible, rather than to impose maximal relational database normalization rules, since the original structure was familiar to users, and limited time was available. In essence this meant that each line class was transformed into a relational table in the new relational database structure, except for Flags which were appended to whichever proto-table(s) they referred, as part of the line instance record they had previously referenced. The compounding of multiple flag values, such as ‘Z2’ meaning stillborn and twin, was left unaltered.

In the Python program, input was processed line by line according to rules specified for each line class. During processing, line classes which shared the same list of variables (such as husbands and wives), were subjected to the same rules, and output as single proto-tables, retaining a line type variable/field for differentiation (for example between spouses who are husbands and those who are wives). Eventually, notwithstanding informational redundancy, in the relational database husbands and wives were placed into separate tables to mirror the original data structure.

FRF reference numbers were retained as the Primary Key to each record, and added to other proto-tables besides the Marriage table as additional variables/fields that would function as Foreign Keys, in order to preserve the relationship between the component line class instances of each record. If duplicate FRF reference numbers were encountered by the program, a warning message was output. Non-atomic variables were converted into separate, atomic variables, so for example baptism date and birth date became separate database fields, and date weightings were given their own fields. NULL values were used where no information was given. Numeric index values starting at 1 were added to Child line instances to track the order in which they appeared in the record, which usually reflects birth order.

The program output files were manually imported into Microsoft Access as database tables, checked, field names were added, and subsequently permanent joins between tables were set up and stored in the database. Joins could also be made using fields named EarlierFRF and LaterFRF in Husband and Wife tables, and OwnFRF in Child tables as Foreign Keys to the FRF Primary Key field in Marriage tables. This represents record linkage to other FRF families belonging to Husband or Wife (which might exist where they were widowed and remarried in the parish), or intergenerationally to FRF families a Child created through its subsequent marriage(s) in adulthood. Such joins were not permanently saved, but described in the database documentation.

Lookup tables were added to the database to define the meaning of coded weighting values and flag values, which had not been fully documented previously, and eventually a lookup for coded occupation values was also provided. These lookup tables are occasionally incomplete or speculative in their definitions where the original meanings, which could be specific to particular family reconstitutions and/or tape punching operatives, had been lost.

Day and month parts of dates that had originally been recorded as 0 were changed to 1. In such cases date weightings were expected to be present to indicate the ambiguity caused by uncertainty in the date value, but in case any such weightings had been omitted and to avoid losing any information, new weighting values were created where such changes had been made. The new weighting in such cases was equal to the old weighting (if any) plus 1,000 for day ambiguity or 2,000 for month ambiguity, since the original weighting values were at most 3 digits in length.

Subsequently the 26 databases were harmonized into one database, by concatenating the records from each table type, so Marriage records from all 26 reconstitution parishes were in one Marriage table, Child records from all 26 reconstitution parishes in one Child table, et cetera.

Besides making all field and table names consistent, the main change on harmonizing the databases into one was to add a new parfrf field that was unique across all 26 parishes. This was derived from the previous FRF Primary Key fields and created by prepending a textual code based on the family reconstitution parish name to copies of numeric FRF field values padded with leading zeroes. This new unique parfrf field replaced FRF as the Foreign Key in other database tables. A further lookup table on the characteristics of the 26 family reconstitution parishes was added to the harmonized database during data deposit with the UK Data Archive in 2018. ^2^

For ease of processing all Child record dates were converted to Access database date type. This data type can only represent valid Gregorian calendar dates. ‘Impossible’ leap year dates of 29 February were changed to 28 February. Where this type conversion entailed changing the date, new weightings were created for the record. These were equal to the old weighting plus 3,000 or 4,000, depending on whether the original date was also invalid in the Julian calendar or not.

After conversion to a relational database the Cambridge Group FRFs are described by nine tables and these main data elements:

- Marriages: Marriage date and location
- Husbands: Name, date of baptism, date of burial, place of birth, occupation, FRF of previous marriage, FRF of next marriage
- Wives: Name, date of baptism, date of burial, place of birth, occupation, FRF of previous marriage, FRF of next marriage
- Children: Name, date of baptism, date of burial, date of marriage, FRF of first marriage
- Husbands’ fathers: Name, residence, occupation
- Husbands’ mothers: Name
- Wives’ fathers: Name, residence, occupation
- Wives’ mothers: Name
- Literacy: Husband’s signature in marriage register, Wife’s signature in marriage register

**Figure 4:**
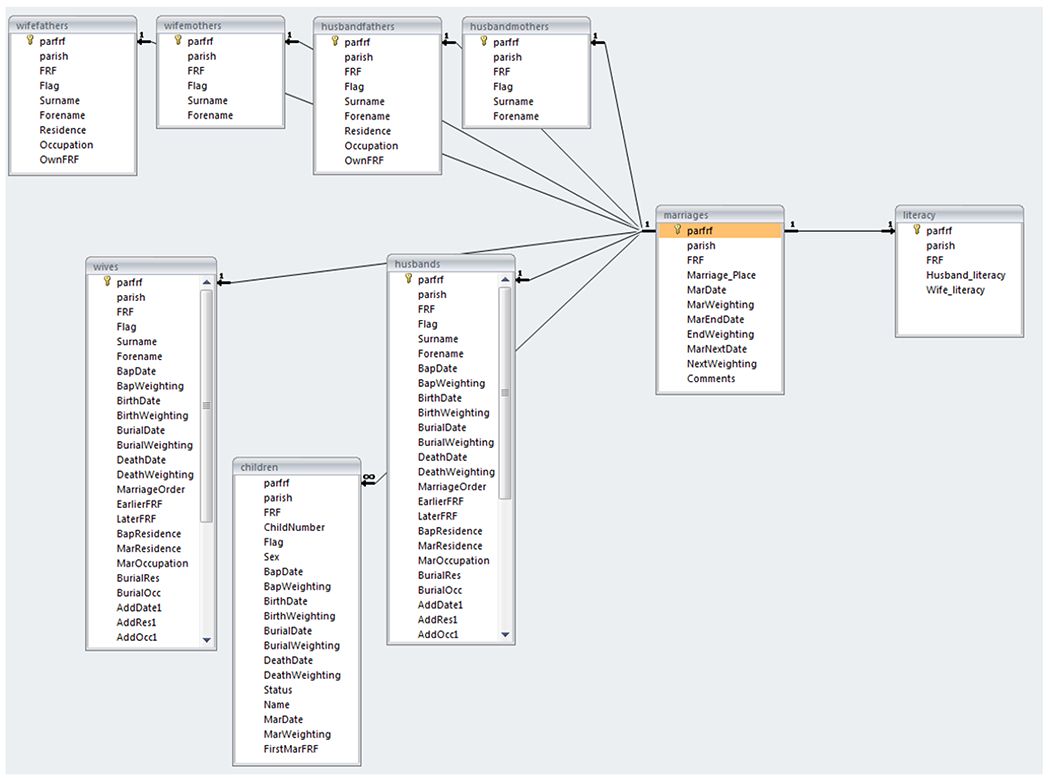
Relational database structure of 26 English parish family reconstitutions dataset

The structure of the relational database tables is shown in Figure 4. This is a screenshot of Access database Relationships, which visualize relational database structure as a simple Entity-Relationship diagram. In the diagram each table/entity is shown as a rectangular box with its name at the top and its fields listed beneath, and joins/relationships between tables are shown as lines drawn between tables’ Foreign Key fields and Primary Key fields. A Primary Key is a unique identifier for a row in a table. A Foreign Key links a row in one table to the Primary Key of a row in another table. Primary Key fields are indicated by a key icon to the left of the field name. The parfrf field described above serves as a Primary Key for marriages. Rows in the Children table are identified by a composite key consisting of the parfrf and the ChildNumber.

The multiplicity of relationships between tables is indicated at either end of the join line in Figure 4. Since a marriage has only one husband and one wife, there are one-to-one relationships between the Marriages table and the Husbands and Wives tables. A row in Marriages may be linked to more than one row in Children, but many marriages have no children in the database. The diagram does not show relationships from one marriage family to another resulting from remarriage, or from a child’s marriage family of origin to its own subsequent marriage(s) as an adult, although these can be made by joining Husbands or Wives EarlierFRF and LaterFRF, or Children FirstMarfrf field, to Marriages table FRF fields within each parish family reconstitution.

### 2.4 CONVERTING THE CAMBRIDGE GROUP RECONSTITUTIONS INTO IDS

Transferring data into IDS (Alter,et al., 2020) involves thinking about data in a different way than working with data in a statistical package, like SPSS or Stata. IDS uses an ‘entity-attribute-value’ model to describe data. In IDS every piece of information or ‘datum’ is a separate record. A record always includes an identifier pointing to an ‘entity’, which is either a person (*ID_I*) or a ‘context’ (*ID_C*). Every record includes a *Type* or *Relation*, designating the kind of information it provides, which may be the date of an event, the *Value* of an ‘attribute’, or the relationship between two people. For example, the following excerpt from an INDIVIDUAL table shows how information about a person is divided across numerous records (rows).

The main steps in transferring the reconstitutions from the relational database into IDS were:

#### 1. Add IDs for individuals and contexts

The nine data tables in the Reconstitutions were linked to each other by a single identifier, *parfrf*, but IDS requires an identifier for every individual. We created individual IDs by adding a role suffix to the *parfrf*.

- Wife: *parfrf* + ‘*W*’
- Husband: *parfrf* + ‘*H*’
- Child: *parfrf* + ‘*C*’ + ‘*xx*’ where *xx* is *ChildNumber*
- Wife’s father: *parfrf* + ‘*WF*’
- Wife’s mother: *parfrf* + ‘*WM*’
- Husband’s father: *parfrf* + ‘*HF*’
- Husband’s mother: *parfrf* + ‘*HM*’

Columns were added to the nine Cambridge Group data tables so that IDs for all of the individuals mentioned in the table were included.

The *parfrf* was also used as a ‘context ID’ for the marital union. Context is a flexible concept that can be used in IDS for any way of grouping people together. A context can be a physical location, such as a house or apartment, or a social grouping, like a household. A context can also be an artifact of the way in which information is arranged in a source document, like a page in a census or population register. An FRF in family reconstitution corresponds to a nuclear family consisting of a husband, wife, and children, and identifying each FRF as a ‘Union’ in IDS is convenient for data extraction software.

#### 2. Births/baptisms and deaths/burials

Most of the original reconstitutions provided dates for baptisms and burials, some gave dates for birth and death, and a small number included both dates. Baptisms were usually a few days after birth, but the delay is sometimes much longer. In a few families, four or five children were baptized on the same day, which probably means that siblings of different ages were baptized together. IDS includes separate types for birth (BIRTH_DATE), baptism (BAPTISM_DATE), death (DEATH_DATE) and burial (FUNERAL_DATE), and all of the dates in the FRFs were included in the IDS file.

#### 3. Convert ‘weightings’ to *Estimations*

Information about the quality of dates in the family reconstitutions was coded into numeric codes called ‘weightings’ in the Cambridge Group (Wrigley et al., 1997, pp. 563–568). For example, if the original document provided the month and year but not the day of a death, the method for computing the day is given in the ‘death weighting’ variable. There are 120 weighting codes in the Cambridge Group family reconstitution database, and the meanings of some codes have been lost. These codes were ordered so that higher numbers indicated less reliability.

Weightings were mapped to the IDS *Estimation* field, which describes the precision of dates. If no weighting is associated with a date, we assume the source provided day, month, and year and designate it as ‘Exact’ in IDS. If only the year was given, we assign an *Estimation* of ‘Estimated (dd/mm)’. Some weightings were translated to ‘Before this date’ or ‘After this date’. For example, sometimes a woman remarried, but the date of death of her first husband is not known. In these cases the date of death of the first husband may be listed as the date of her second marriage with *Estimation* as ‘Before this date’. (See also Wrigley et al. (1997, p. 576) for inferring the father’s date of death from a birth recorded as ‘posthumous’.)

#### 4. Convert ‘flags’ to *Types*

‘Flags’ were used for various types of less common information. There are flags for widowhood, religious denominations, illegitimacy, and paupers. Flags were translated into IDS information *Types*. For example, senior/junior and elder/younger were recorded in IDS *Type* ‘SUFFIX_NAME’. Columns were added to the nine Cambridge Group data tables for Pauper, Posthumous, Name suffix, and Religion, because these *Types* could not be recorded in existing columns. We did not transcribe flags when they could be inferred from information available elsewhere in the database, such as marital status at death.

#### 5. Map columns to IDS *Types* and IDS Transposer

The IDS Transposer is a web service that converts rectangular data files into IDS format files (Klancher Merchant & Alter, 2017). The IDS Transposer requires two ‘mapping’ files describing how the original data files will be represented in IDS. We give a brief overview here and refer readers to Klancher Merchant and Alter (2017) for advice on the construction of the mapping files.

The Entity mapping file associates columns in the input files with attributes and events in the IDS INDIVIDUAL and CONTEXT tables. Each IDS record must have a *Type* identifying the kind of information it contains and an ID pointing to an individual or a context. INDIVIDUAL and CONTEXT records may have a *Value* (such as ‘male’ or ‘female’ for *Type*=Sex), and most records have a timestamp showing when an event occurred or the attribute was observed.

The Relationship mapping file controls the creation of records for the INDIV_INDIV, INDIV_CONTEXT, and CONTEXT_CONTEXT tables. These tables describe a *Relation* between two entities, i.e. individuals or contexts. Relationships in the INDIV_INDIV table are always given in both directions, e.g. ‘mother of’ and ‘child of’ (Alter & Mandemakers, 2014).

As mentioned above, we consider each FRF a marital union. A record was created for each FRF in the CONTEXT table with *Type*=‘Union’ and timestamp set to the date of marriage. Each person in the FRF was linked to the Union in the INDIV_CONTEXT table with a relation of ‘Wife’, ‘Husband’, or ‘Child’.

Most of the work of transferring data from the nine tables in the Cambridge Group reconstitutions database to the five IDS tables was done by the IDS Transposer, but several additional processing steps were needed.

#### 6. Harmonize individual IDs

Wherever possible, individuals appearing in more than one FRF were assigned the same ID everywhere in the IDS database. References linking individuals across FRFs are available in the original database. The Children table includes a column showing the FRF of the first marriage of that child, and the Husbands and Wives tables have columns for previous and next marriages. These references were used to create a table of equivalent IDs, and individuals were assigned the ID that occurred earliest in time in the database.

#### 7. Add parish-level context information

We use the CONTEXT table to add information that is common to every FRF in a parish, such as county, ‘quality’ years, and Sample Groups, which are discussed below. Since IDS uses a relational model, ‘quality’ years and Sample Groups are recorded once in the CONTEXT table and linked to each FRF through a ‘Union in parish’ relationship in the CONTEXT_CONTEXT table.

#### 8. Other processing

A number of other minor changes were made to the tables produced by the IDS Transposer. In some situations, the IDS Transposer created empty records that were deleted. We also corrected some errors in the database.

We ran a suite of error checks on the IDS database to detect inconsistencies, such as death dates that precede birth or marriage dates or births to mothers younger than 15 or older than 50. A list of these known problems is available with the IDS database.

### 2.5 FROM IDS TO EPISODES

Data in IDS format must be converted to a rectangular data array for analysis by standard statistical packages. Longitudinal data described in IDS needs to be transformed into episodes — segments of time in a fertility history. Each episode has start and end dates. Episodes that do not end in a birth are considered ‘right censored’ in event history analysis. Episodes are divided (left and right truncated) when a variable changes, so that all explanatory variables are constant within an episode.

We use a two-step process developed by Luciana Quaranta to move from IDS to an episodes file (Quaranta, 2015, 2016). The first step is creating a ‘Chronicle’ file from the IDS. The Chronicle file provides a life history in the form of dates when individual or contextual attributes changed. For example, we know that the value of marital status becomes ‘single’ on a person’s birthdate, ‘married’ on their marriage date, and ‘widowed’ on the date of death of their spouse. If the database includes declarations of marital status from a census or population register, the marital statuses recorded on those dates can also be included. The Chronicle file also identifies the occurrence of the event of interest (e.g. childbirth).

A Chronicle file uses an ‘entity-attribute-value’ approach very similar in structure to IDS, but the attributes may be complex time-varying measures, such as ‘number of surviving sons’. Attributes in the Chronicle file will become variables in the Episode file used for analysis. We created variables that may be useful for analyzing fertility, such as date of death of the preceding child and an indicator for lactation. The Chronicle file was created from the IDS tables using SQL in Microsoft Access.

The Chronicle file was imported into a Stata file, which was converted to episodes by a modified version of Quaranta’s Episode File Creator script (Quaranta, 2015, 2016). The EpisodesFileCreator is sensitive to duplicate events and other problems in the chronicle file. Duplicate events often point to errors in the data, but the Chronicle file must be designed to resolve conflicts when events occurred on the same day, like twin births. Changes to the EpisodesFileCreator.do file were designed to help in debugging the Chronicle file. In several places temporary variables or files are saved to make it easier to find problems in the Chronicle file. See the section titled ‘Access to Data and Program Code’ for access to the programs and scripts used to create the Chronicle and Episode.

### 2.6 DATA QUALITY TESTS

IDS is particularly useful for performing tests on the consistency of information in a database. The relational structure of IDS makes it easy to compare dates within individual histories and between related individuals. We ran about a dozen tests of this kind, and the CAMPOP data are remarkably clean.^3^ We found only two cases where the vital events (birth, marriage, death) in an individual’s life were out of order. We found no cases where a mother is less than age 15 or older than age 50 at the birth of a child, and less than one percent of fathers were younger than age 18 or older than 60. The very small proportion of families with inconsistent data is testimony to the care taken in preparing the CAMPOP database. Although the reconstitution of families was done by sorting pieces of paper, the Cambridge group tested the data extensively after it was digitized (Wrigley et al., 1997, pp. 574–577).

It is important to remember that the parish registers report dates of baptisms and burials rather than dates of births and deaths. Wrigley et al. (1997, p. 111) note that baptisms were usually performed quickly, but the intervals between birth and baptism widened over time. By the late 18th century there were long lags between birth and baptism in some parishes. The IDS database includes 556 (about 0.2%) intervals between baptisms that are greater than 0 and less than 250 days.

### 2.7 DIFFERENCES BETWEEN THE IDS DATA AND PUBLISHED RESULTS

Fertility calculations using the IDS file and associated programs will differ from the numbers presented in Wrigley et al. (1997). We have determined that the main source of differences is our treatment of flags for widows/widowers in the original FRFs. In some parishes these flags were used to select which families were included in the fertility analysis when a burial date was available for only one spouse. These flags may reflect information in the burial registers, in which women were sometimes recorded as widows. But some of these notations on the FRFs were made after the family had been reconstituted. When we compared the ‘W’ flags to the burial dates in the FRFs, we found discrepancies that we cannot explain. Unfortunately, the researchers who worked on the reconstitution volume had information about the quality of these notations that is no longer available. For this reason, we have chosen to ignore the ‘W’ flags and not to include them in the IDS database. We only use the dates of burial included in the data to infer widowhood and the end dates of marriages. This results in small differences between our estimates of fertility rates and those in the reconstitution volume.

## 3 SELECTING FAMILIES FOR FERTILITY ANALYSIS

Wrigley et al. (1997, pp. 617–622) devoted an appendix to explaining the criteria used for selecting which families were used in different types of fertility analysis.^4^ In this section we ask which of these criteria have important effects on results. Some selection criteria have very little impact on results, but others can bias results in important ways. Unfortunately, some recent publications do not follow important rules for analyzing family reconstitutions, and they include results that are biased and incorrect (see critiques found in Alter, 2019; Clark, Cummins, & Curtis, 2019).

Family reconstitution is inherently the study of the geographically stable part of a population. Following the model pioneered by Louis Henry and his colleagues, the CAMPOP reconstitutions describe individual parishes. Family histories of migrants are incomplete and excluded from analysis, because events that occurred in other parishes are not available. Steven Ruggles (1992) argues that this focus on the immobile part of the population biases estimates of marriage ages and mortality based on family reconstitutions. Although Ruggles (1992, pp. 522, note 517) suggests that estimates of fertility may be affected by the exclusion of migrants, Henry (1976) and Wrigley (1997) conclude that marital fertility did not differ significantly between movers and stayers in rural areas. We explain below why migrants are excluded by the logic of family reconstitution.

### 3.1 REFERENCE MARITAL FERTILITY FILE

We examine the effects of different selection criteria on fertility by comparing them to marital fertility rates computed from a Reference sample. Criteria for inclusion in the Reference sample are in the box below. Unless otherwise specified, birthdate requirements may be met by actual birth dates or proxy baptism dates, and similarly death date requirements may be met by actual death dates or proxy burial dates. We will discuss each group of criteria and compare marital fertility rates from alternatives to the rates in the Reference sample.

The fertility analysis below treats multiple births as a single event. This has practical advantages, because the Stata procedures that we use are not designed to handle simultaneous events. We also classify all baptisms within 60 days as multiple births. Twins might be baptized at different times, because infants considered weak and vulnerable were often baptized early, and the delay between birth and baptism increased in the 18th century (Wrigley et al., 1997, p. 473). Multiple births are about 1.5% of all births, and the marital fertility rates given here can be inflated by that amount for comparison with other sources.

#### 3.1.1 CRITERIA 1 — PRE-REQUISITES: REQUIRED FOR COMPUTING AGE-SPECIFIC MARITAL FERTILITY RATES

These pre-requisites are minimal criteria for computing age-specific marital fertility rates, but Table 1 shows that they exclude about 60% of the data in the family reconstitutions. This is not unusual or unexpected. The main problem is lack of a birthdate for the mother. Since ages at marriage were not usually reported in the parish registers of pre-industrial England, the only way to know a mother’s age is to find her baptism. This automatically excludes all women born before the parish registers began. It also excludes all women who were born in a different parish.

#### 3.1.2 CRITERIA 2 — HENRY REQUIREMENTS

Family reconstitution solves a fundamental problem for demographic analysis of vital events in the period before censuses. Louis Henry showed that family histories could be used to measure time at risk of demographic events, but he provided strict rules about which family histories can be included in each kind of computation (Fleury & Henry, 1985; Henry, 1970; Henry & Blum, 1988). Henry’s rules are designed to avoid ‘informative censoring’, which biases the computation of demographic rates. For example, the family history must include the death of either the husband or wife to demonstrate that the couple resided in the parish during all of their marriage. However, the requirements for some computations, like average birth interval length, can only be applied to families where both spouses were alive when the wife reached age 50.

The Cambridge Group strictly adhered to Henry’s rules. But, unfortunately, some recent authors have ignored those principles under the incorrect assumption that statistical methods can compensate for biased data (Cinnirella, Klemp, & Weisdorf, 2017; Van Bavel, 2004a; Van Bavel, 2004b; Van Bavel & Kok, 2004). We show here that ignoring Henry’s guidelines results in serious biases. For a discussion of the impact of these biases on event history models, such as Cox proportional hazards, see Alter (2019).

The central problem in family reconstitution is determining when a couple was resident in the parish being studied. Since the parish registers do not record migration, we only know that a couple was present in the parish when an event (baptism, marriage, burial) occurred. This is known in the statistical literature as Passive Registration (see Gill, 1997). However, only the death of the husband or wife can be used to mark the end of a family history for the purpose of studying fertility. Although the birth or death of a child also shows that the family was residing in the parish, the rules of family reconstitution exclude family histories that end with these child-related events. Fleury and Henry provide a very brief justification for this rule:

“As the date of the end of union holds an essential place in the study of fertility, it is only usable when it is known independently of any document, death or marriage in particular, concerning the children; not respecting this rule favors the most fertile families and leads to an over-estimation of fertility (Fleury & Henry, 1956, p. 183; author’s translation).”

When a family history ends with the birth of a child, there was some period of time between the last observed birth and their departure from the parish. This period should be included in the denominator of the fertility rate, because the woman was at risk of a birth during this time. Since we have no way of knowing how much time elapsed between the last birth and the family’s departure, including this family in our computation will overestimate the fertility rate. In addition, the time between the last observed birth and migration will be shorter for women who have shorter birth intervals, and ending fertility histories with the last observed birth will capture more births from women with higher fertility.

Fleury and Henry were describing a principle now known as ‘non-informative censoring’ in the statistical literature (Kalbfleisch & Prentice, 1980, pp. 195–196). When the time used to end an event history is related to the event of interest (e.g, a birth), time at risk is underestimated and transition rates are overestimated (see Wrigley et al. (1997, pp. 12–17) for a discussion of the logic behind the Henry rules for family reconstitution).

**Figure 7:**
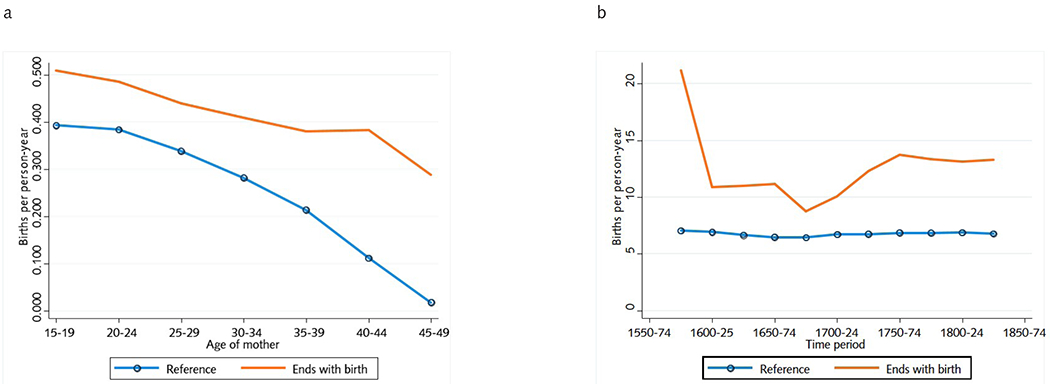
a *Effect of Henry Requirements on Age-specific Marital Fertility Rates* b *Effect of Henry Requirements on Total Marital Fertility Rate (Ages 20–49) by Period*

As expected, fertility is overestimated in Cambridge Group family histories that violate Henry’s requirements. Figures 7a and 7b compare the Reference sample to families excluded because their histories end with a birth or the death of a child under age 15. Figure 7a shows that the bias is especially large at older ages. Very few women gave birth after age 45, but the family histories excluded by Henry always include a birth. So, the bias in fertility rates excluded by the Henry rules varies by age.

Figure 7b shows that total marital fertility rates for periods are also biased when the Henry rules are violated. Movements in fertility among family histories ending with a birth are an artifact of the composition of the data and do not correlate with the movements of fertility in the Reference sample.

**Figure 8:**
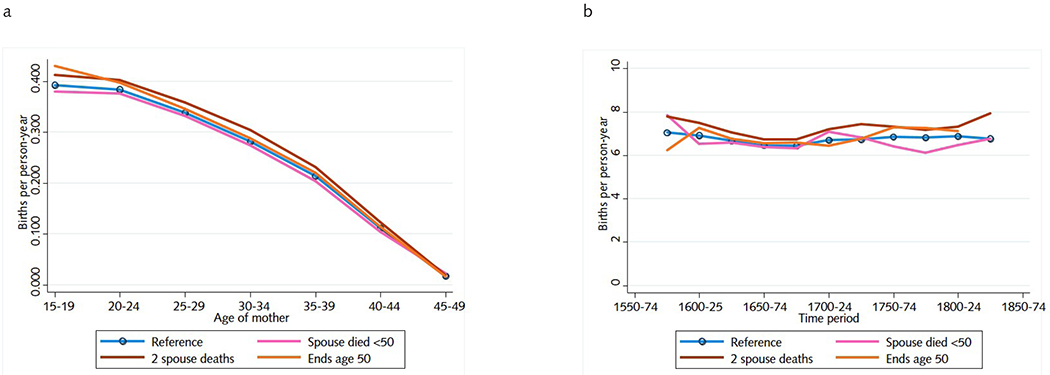
a *Effect of Spouse Death Rules on Age-specific Marital Fertility Rates* b *Effect of Spouse Death Rules on Total Marital Fertility Rate (Ages 20–49) by Period*

The Henry rules also specify more strict selection rules for special types of analyses. In particular, average durations of birth intervals are only computed from marriages that continued until the wife reached age 50. In Figures 8a and 8b we examine different variants of the Henry rules of family reconstitution. ‘Ends age 50’ includes only marriages in which the first spouse death occurred after the wife reached age 50. ‘Spouse died <50’ are the marriages interrupted before the wife reached the end of her reproductive years. ‘Two spouse deaths’ is a stricter version of the Henry rules in which we only include couples where dates of death are available for both spouses.

The results of these comparisons are reassuring. Age-specific marital fertility rates computed under these criteria differ little from the Reference sample, except at the youngest ages. Marriages that survived until the wife reached age 50 had slightly higher fertility than marriages ended by death before age 50, which may be a reflection of differences in health.

#### 3.1.3 CRITERIA 3 — PRECISION OF DATES

**Figure 9:**
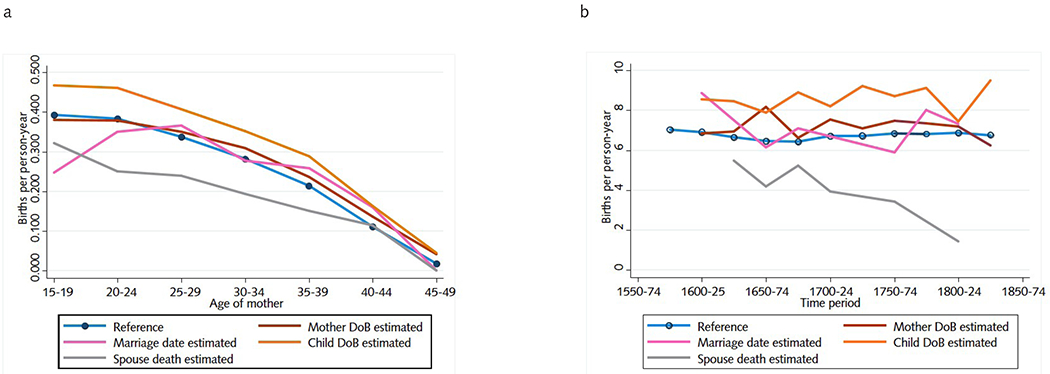
a *Effect of Date Precision Requirements on Age-specific Marital Fertility Rates* b *Effect of Date Precision Requirements on Total Marital Fertility Rate (Ages 20–49) by Period*

The Reference sample follows the practice in Wrigley et al. (1997) of excluding families when the month of occurrence of an important event is estimated. Figures 9a and 9b show the consequences for computing marital fertility when dates of marriage, mother’s birth, spouse death, and children’s births have been estimated.

Families in which the mother’s date of birth or the date of marriage have been estimated do not appear to be systematically different from the Reference sample. The age patterns of marital fertility for these families are similar to the Reference sample, except for women ages 15–19 with estimated marriage dates (Figure 9a). Deviations from the Reference sample by time period (Figure 9b) are likely due to small numbers. There are only 692 person-years and 171 births for couples whose month or year of marriage was estimated.

Fertility calculations for couples without a precise date for the death of a spouse are substantially different from the Reference sample. Fertility appears to be much lower among these couples and their fertility seemingly decreases over time. These apparent features are probably due to dates of death estimated from the remarriage of a surviving spouse. If a husband or wife remarried but the death of their previous spouse was not observed, the Cambridge Group reconstitutions sometimes use the remarriage date with a flag saying ‘before this date’. Using these dates in fertility calculations overestimates the duration of the first marriage and underestimates marital fertility rates.

The Reference sample is restricted to family histories in which the month of birth of a child is known, which is a less strict criterion than we use for other dates. We relaxed this criterion to include families where a birth was inferred from a burial record without a matching baptism (Wrigley et al., 1997, pp. 110–112). This situation was most likely to occur when a child died before being baptized. When these burials could be linked to the parents’ FRF, a ‘dummy’ birth record was created with the date of burial used as the estimated birth date.

Figures 9a and 9b show that fertility rates calculated for families with at least one estimated date of birth are higher than those in the Reference sample. This should be expected, because families with higher fertility had more chances for a child to die without a baptism. However, it does suggest that the Reference sample may be underestimating marital fertility slightly. If we relax the criterion for precision of birth dates to allow uncertainty about the month of birth, the total marital fertility rate for ages 20–49 increases by about 1.5% from 6.7 to 6.8.

#### 3.1.4 CRITERIA 4 — FIRST MARRIAGE FOR BOTH HUSBAND AND WIFE

**Figure 10:**
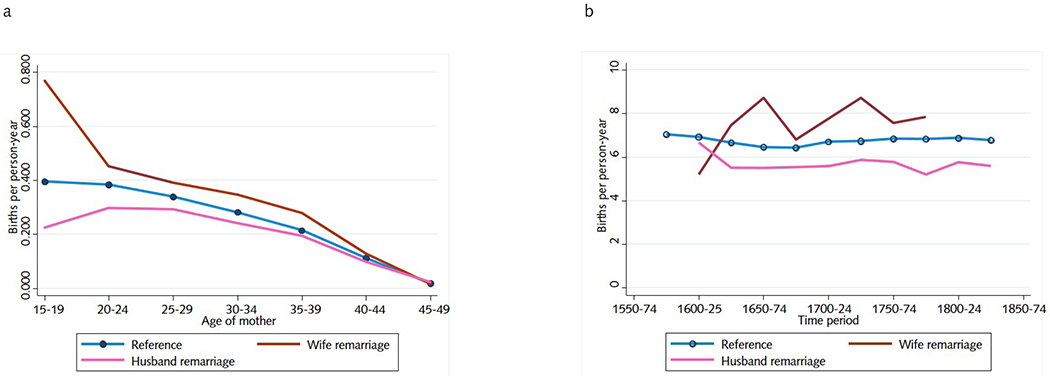
a *Effect of Remarriage on Age-specific Marital Fertility Rates* b *Effect of Remarriage on Total Marital Fertility Rate (Ages* *20–49) by Period*

A common practice in historical demography is to focus on marital fertility in bachelor-spinster marriages. Figures 10a and 10b show that fertility was somewhat different in remarriages. The Reference sample consists of first marriages for both husband and wife, and we compare those families to marriages in which a widow remarried (regardless of the marriage order of the husband) and those in which a widower married a never married woman. The fertility of remarried widows was higher than the Reference sample, and the fertility of remarried widowers was lower than the Reference sample. The biggest discrepancies occur at ages 15–19 where premarital pregnancies were more common in widow remarriages and less common in widower remarriages, but the number of cases was small.

#### 3.1.5 CRITERIA 5 — CAMPOP SELECTION CRITERIA

**Figure 11:**
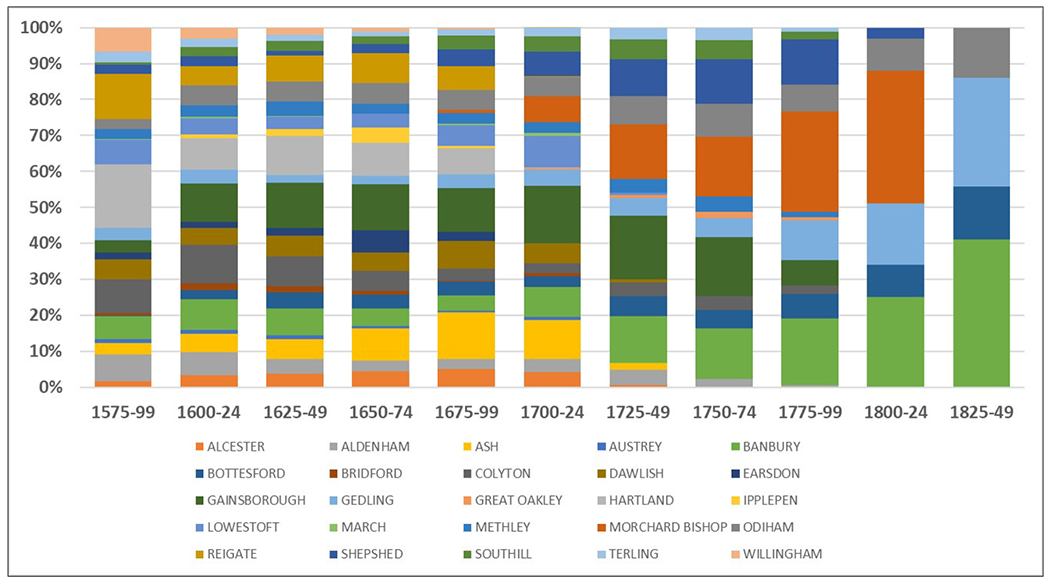
Percent of Person-years at Risk by Parish and Period, 1575–1849 See Appendix Table 1 for numbers of person-years by period in each parish.

The 26 family reconstitution studies used in the 1997 volume were the best data available, but they were not randomly or systematically selected. Parishes were assigned to four ‘sample groups’ to approximate a representative sample of England. The composition of these groups changed over time to compensate for geographic imbalances in the availability of data (Wrigley et al., 1997, pp. 40–72). Figure 11 shows the changing contributions of parishes to time at risk by time period.

Two parishes received special treatment in our analysis. The parish of Birstall was omitted from calculations of marital fertility rates, because FRFs were not created for families with no children (Wrigley et al., 1997, p. 356). Inclusion of Birstall would bias fertility rates, but Birstall is used for some analyses not involving childless couples. When Birstall is included, it is given half weight because of its large population. The parish of Shepshed, which had a high concentration of manufacturing, is given half the weight of other parishes to better approximate the occupational structure of England (Wrigley et al., 1997, pp. 42–48).

The data for each parish were divided into time periods by both ‘quality’ and ‘sample groups’. ‘Quality’ reflects the evaluation of Cambridge Group researchers of the completeness of reporting of events in a parish. A variety of tests were used to evaluate the reliability of the data (Wrigley et al., 1997, pp. 73–118), and periods of high quality recording were assigned to each parish. ‘Groups’ were defined as subsets of parishes and years within which composition effects were minimized, making within-group comparisons plausible.

**Figure 12:**
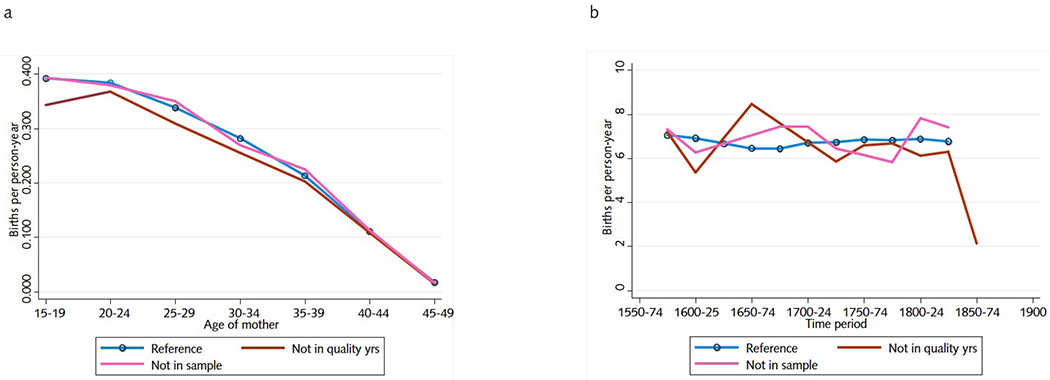
a *Effect of ‘Quality’ and Sample Years Rule on Age-specific Marital Fertility Rates* b *Effect of ‘Quality’ and Sample Years Rule on Total Marital Fertility Rate (Ages 20–49) by Period*

Figures 12a and 12b compare the Reference sample to family histories excluded because they began or ended outside of ‘quality’ years or ‘sample groups’. These results confirm the importance of both selection criteria. Fertility rates calculated on years not designated as ‘quality’ fall below the Reference sample at all ages and most periods indicating that births were under-reported during these years. Families dropped for not appearing in a sample had the same age-specific marital fertility rates as those in the Reference sample, but total marital fertility rates would follow a different pattern over time if they were included.

### 3.2 AVAILABILITY OF OCCUPATION

Occupations are reported in the marriage section of the FRFs, and we assume that occupations refer to the date of the marriage. Among the 68,000 husbands in the entire database, occupations are given for 36,000. We have occupations for less than 2,700 wives. Marriages sometimes gave occupations for fathers of the bride (10,000 observations) and the groom (11,000 observations).

**Figure 13:**
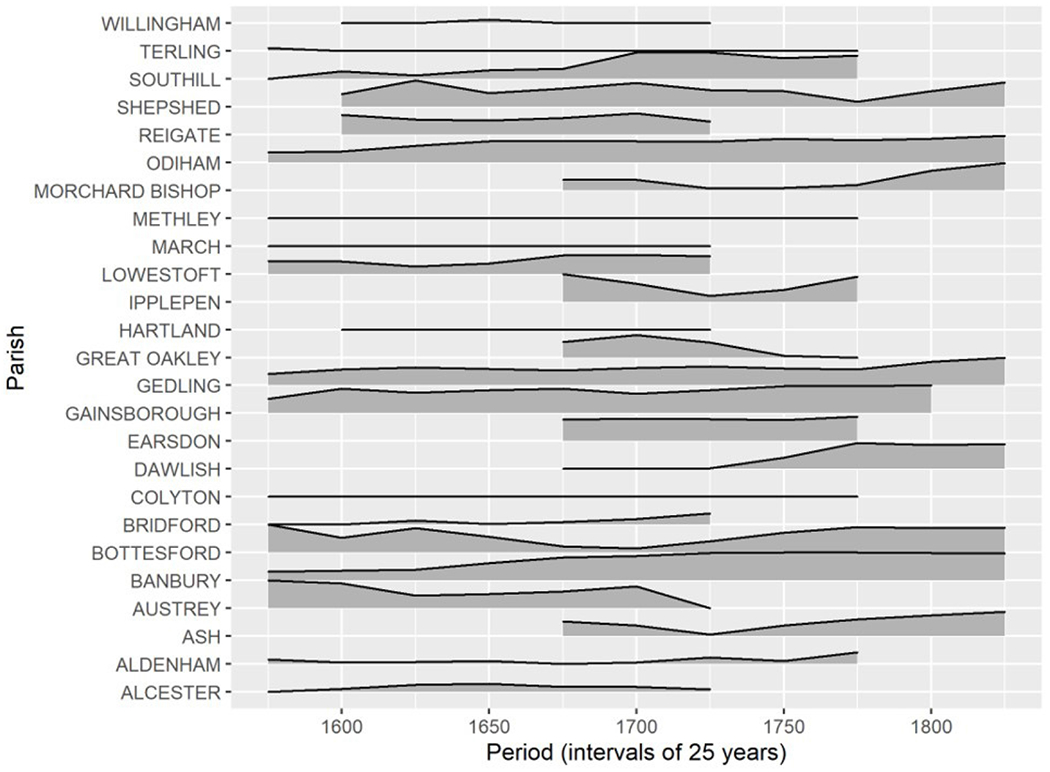
Proportion of Husbands with Occupations by Parish and Time Period

Occupations are available for about half of the family histories in the Reference sample. However, the availability of occupations varies considerably by parish and time period. As Table 2 shows occupations are available for more than 70% of the husbands in some parishes, but four parishes have no occupations at all. Figure 13 shows when husbands’ occupations were available in each parish. Occupations were more likely to be available after 1700, but in several parishes, like Bottesford and Morchard Bishop, recording of occupations decreased and then increased. This means that any analysis that uses occupation as a covariate may be subject to marked selection and composition effects.

**Figure 14:**
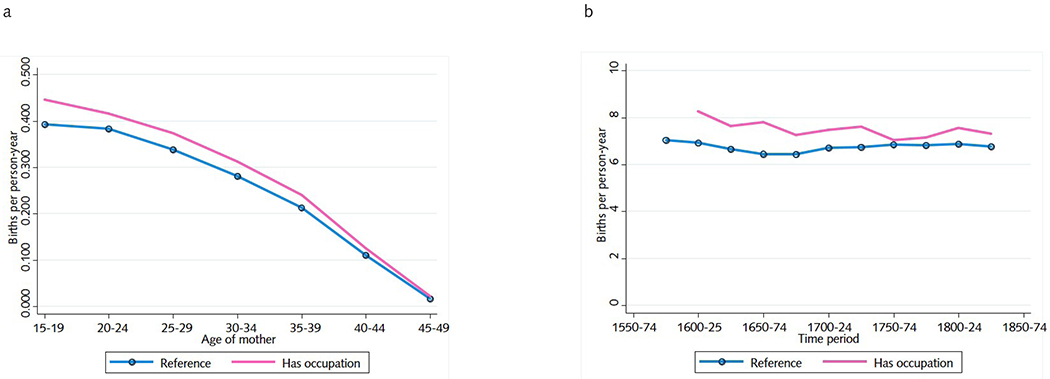
a *Effect of Availability of Husband’s Occupation on Age-specific Marital Fertility Rates* b *Effect of Availability of Husband’s Occupation on Total* *Marital Fertility Rate (Ages 20–49) by Period*

Changes in availability of occupations (Figure 13) affect estimates of marital fertility shown in Figures 14a and 14b. Compared to the Reference sample, fertility histories with occupations had higher age-specific fertility, and the difference is greater before 1750.

## 4 CONCLUSIONS

We have explored how the Cambridge Group family reconstitutions were originally constituted for demographic use and have evolved as a dataset. They are now used by other researchers in disciplines where family reconstitution is an unfamiliar method and source. For this wider audience this paper has elucidated how the composition of these data and choices made in using them will affect, and potentially bias, outcomes. As test cases to demonstrate this principle we have replicated and extended analyses of marital fertility, showing the effect that segmenting the data in various ways has on observable results and their validity. We have also probed underlying geographical and chronological variations in coverage. This serves to underline differences in how universally available are variables such as occupation, and we have also detailed the importance of bearing in mind the extent and precision with which core dates establishing the presence or absence of each family from analysis, such as the marriage date, are knowable or were originally recorded.

Our objective has been to provide a deeper understanding of the Cambridge Group family reconstitutions that can guide future research. We strongly believe that these data are a rich resource for both historical and demographic research, and we hope that providing the data in new formats (IDS, episodes) will encourage new types of analysis. However, we emphasize the importance of basing future research on sound methods and appreciation of the underlying sources. The Cambridge Group spent a great deal of time and effort detecting and understanding problems in these data. We strongly encourage researchers to rely on the variables, like ‘sample years’ and ‘quality periods’ which are included in the data. We also emphasize that the principles of family reconstitution must be understood and respected.

We have shown that analyses of these data are very sensitive to decisions about the selection of families for analysis. In particular, informative censoring (i.e., including life histories with an event correlated with the transition of interest) biases calculations of demographic measures in a predictable way. For example, fertility histories ending with the birth or death of a child must be excluded from analysis, because they underestimate birth rates. Henry, Wrigley, Schofield, and the other pioneers of historical demography understood this problem well. They based the rules of family reconstitution on fundamental insights about data analysis that are as important for sophisticated statistical models today as they were fifty years ago.

## 5 ACCESS TO DATA AND PROGRAM CODE

Data and program code used in this article are available from public data repositories. The complete database of family reconstitutions of 26 parishes is available from the UK Data Service. See:

Wrigley, E. A., Davies, R. S., Oeppen, J. E., & Schofield, R. S. (2018). *26 English parish family reconstitutions* [Data collection]. Colchester, Essex: UK Data Archive. 10.5255/UKDA-SN-853082

The IDS version of the data is available from:

Alter, G., Newton, G., Oeppen, J., Wrigley, E.A., Davies, R., Schofield, R. (2020). CAMPOP: 26 English family reconstitutions in intermediate data structure format with fertility analysis files 1538-1851. [Data collection]. Colchester, Essex: UK Data Service. 10.5255/UKDA-SN-854465

IDS data are in comma-separated text files. We also provide episode files for studying marital fertility in Stata format (.dta) for the Reference sample and five other samples described above. The fertility files include a number of variables not discussed in this article.

Program code used in this article is available from the OpenICPSR repository. See:

Alter, G. (2020–08–11). *Re-introducing the Cambridge Group Family Reconstitutions.* Ann Arbor, MI: Inter-university Consortium for Political and Social Research [distributor]. https://doi.org/10.3886/E120585V1

These programs begin with the IDS database and produce all of the samples and analyses discussed here. A Readme file is included that describes the steps in moving from IDS to fertility analysis episode files. The first step is a Microsoft Access database that uses SQL queries to create the Chronicle file, which is then translated to a Stata dta file. A slightly modified version of Quaranta’s (2016) Episode File Creator is used to convert the Chronicle file into an episode file. Several Stata scripts (do-files) prepare the data, partition episodes by age group and time period, and compute graphs and tables. Fifteen Stata do files extract samples from the episode file under different selection criteria.

## Figures and Tables

**Figure 5:**
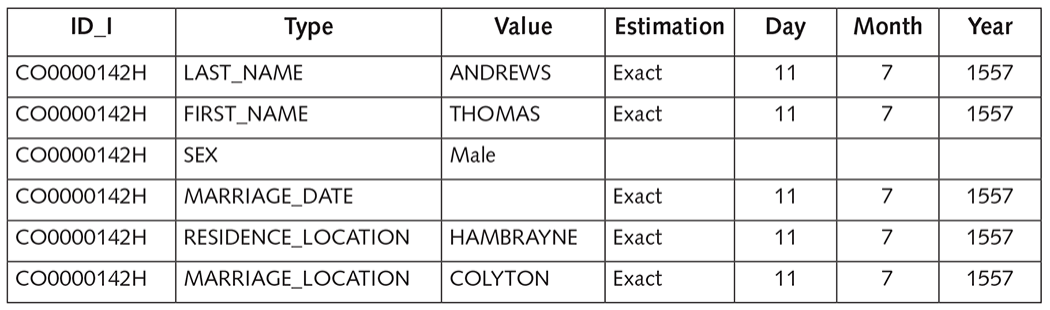
Example of IDS INDIVIDUAL Table

**Figure 6:**
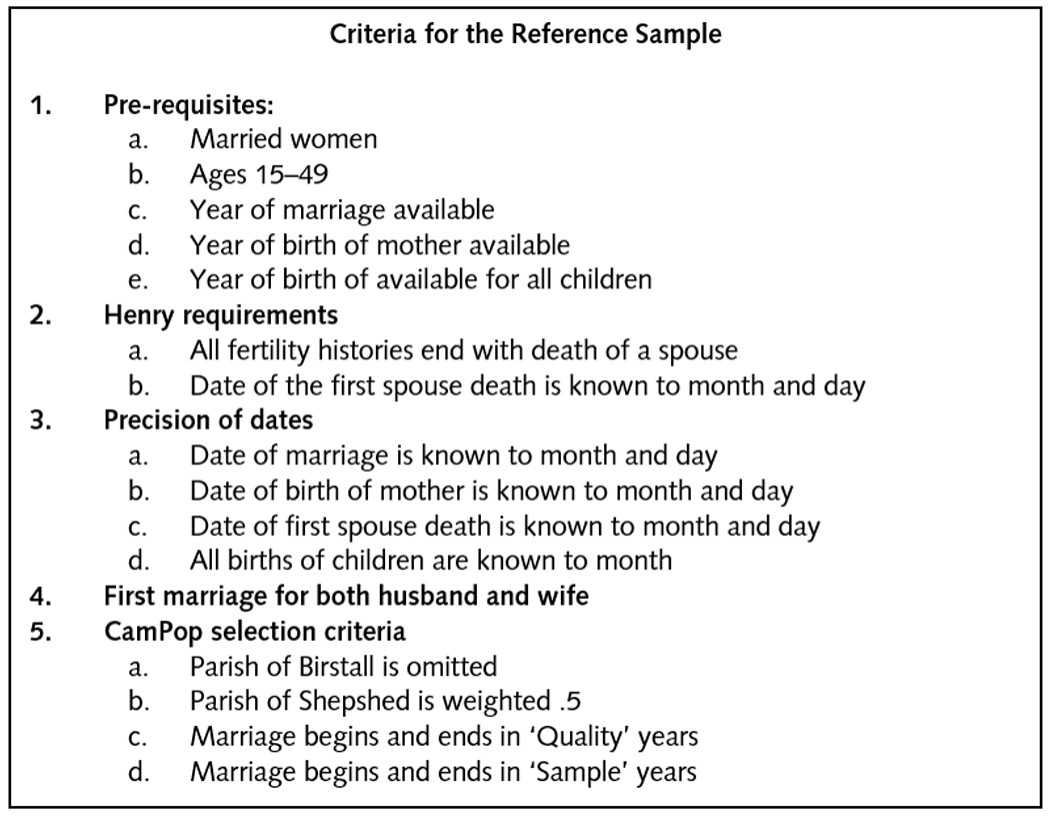
Criteria for the Reference Sample

**Table 1:** Number of Births and Person-years of Observation by Pre-requisite Sample Selection Criteria

	Births		Person-years	
	Number	Percent	Number	Percent
Marriages with at least one event and no undated births	132,005	100%	666,470.0	100%
Marriages satisfying pre-requisites	57,538	44%	269,576.1	40%
Reference sample	22,653	17%	118,856.6	18%

**Table 2:** Percent of Births and Person-years in Family Histories with an Occupation for the Husband by Parish

Parish	Births	Person-years
Alcester	21%	18%
Aldenham	11%	6%
Ash	50%	47%
Austrey	75%	63%
Banbury	82%	79%
Bottesford	63%	57%
Bridford	8%	6%
Colyton	0%	0%
Dawlish	51%	45%
Earsdon	82%	73%
Gainsborough	86%	77%
Gedling	69%	63%
Great Oakley	31%	21%
Hartland	0%	0%
Ipplepen	44%	39%
Lowestoft	55%	50%
March	0%	0%
Methley	0%	0%
Morchard Bishop	32%	28%
Odiham	75%	69%
Reigate	63%	57%
Shepshed	59%	48%
Southill	63%	60%
Terling	1%	1%
Willingham	4%	3%
		
Total	52%	46%

## Footnotes

1 Paper tape is an early data storage medium, using perforations for binary encoding of data. It was eventually superseded by magnetic tape.

2 UK Data Archive Study Number 853082, available at: http://doi.org/10.5255/UKDA-SN-853082.

3 A small number of records were edited by Alter to solve problems detected during the creation of the IDS data tables. In most cases these were obvious transcription errors or dates that could be corrected from other information in the database.

4 In the following discussion we use the term ‘family’ to refer to the conjugal or nuclear family, which is the group described by an FRF.

## Appendix

**APPENDIX TABLE 1 —:** NUMBER OF PERSON-YEARS AT RISK BY PARISH AND TIME PERIOD, 1575–1849

	Time period										
Parish	1575–1599	1600–1624	1625–1649	1650–1674	1675–1699	1700–1724	1725–1749	1750–1774	1775–1799	1800–1824	1825–1849
Alcester	55.5	371.4	532.9	578.8	589.3	474.4	61.3				
Aldenham	233.1	699.6	562.4	369.1	323.9	414.2	433.5	262.5	42.2		
Ash					103.7	546.4	774.8	1153.4	1510.2	1227.3	198.2
Austrey	37.6	122.7	152.1	87.3	45.7	76.5	0.4				
Banbury	205.1	931.3	1056.2	644.5	501.9	934.2	1357.2	1625.2	1641.2	1479.0	275.0
Bottesford	1.6	259.1	616.4	475.7	450.1	352.5	555.4	598.4	599.0	533.4	98.6
Bridford	20.6	221.9	236.1	146.7	32.7	97.2	12.9				
Colyton	300.7	1157.7	1152.4	719.0	384.9	314.6	396.0	452.8	191.9		
Dawlish					181.0	505.2	808.5	670.2	902.8	620.8	92.1
Earsdon					58.6	177.4	301.1	781.0	294.1		
Gainsborough	107.4	1159.5	1757.0	1659.4	1423.5	1793.7	1807.3	1905.3	631.5	0.6	
Gedling	114.4	431.2	315.7	301.4	444.9	504.4	516.2	622.8	959.7	1003.8	203.5
Great Oakley					7.8	47.8	92.5	175.9	77.0		
Hartland		559.7	958.6	1509.7	1192.1	827.0	18.8				
Ipplepen					0.6	110.2	269.9	529.2	81.1		
Lowestoft	219.3	475.1	480.3	496.1	673.6	995.6	52.9				
March	5.3	44.2	50.5	19.6	43.7	83.7	4.2				
Methley	93.3	362.4	550.7	349.6	349.2	333.7	394.1	505.0	142.6		
Morchard Bishop					103.7	822.8	1567.8	1934.8	2443.4	2171.2	365.7
Odiham	84.7	592.0	772.0	736.4	640.9	597.7	826.5	1067.5	659.0	529.0	92.8
Reigate		405.6	596.9	1020.7	1066.6	770.4	36.2				
Shepshed		77.8	288.3	182.1	336.8	552.6	776.0	1037.1	1425.8	1096.6	177.8
Southill	24.4	277.3	375.0	287.6	438.5	472.1	573.8	609.4	180.3		
Terling	94.0	255.4	249.9	152.1	208.6	267.0	345.6	408.3	105.1		
Willingham		214.3	335.5	279.6	148.7	53.4	1.3				
